# Corrigendum

**DOI:** 10.1111/jdi.12295

**Published:** 2015-01-01

The publisher would like to draw the reader's attention to the errors in the following article.

Lee EY, Hwang S, Lee SH, Lee Y-h, Choi AR, Lee Y, Lee B-W, Kang ES, Ahn CW, Cha BS, Lee HC. Postprandial C-peptide to glucose ratio as a predictor of β-cell function and its usefulness for staged management of type 2 diabetes. *J Diabetes Invest* 2014; 5: 517–524.

On page 519 and 520, ΔCP should have been defined as delta C-peptide in the footnote of Table 1 and 2 respectively.

On page 521, abbreviation for fasting C-peptide-to-glucose ratio should have been FCGR in the footnote of Table 3.

The publisher apologizes for the errors and for any confusion caused.

